# A cross-sectional study to ascertain malaria prevalence among asymptomatic travellers arriving on the Lihir Group of Islands, Papua New Guinea: implications for elimination efforts

**DOI:** 10.1186/s12936-023-04804-y

**Published:** 2023-11-29

**Authors:** Pere Millat-Martínez, Bàrbara Baro, Bernadine Kasian, Lina Lorry, Sergi Sanz, Chilaka Wali, Sylvia Raulo, Arthur Elizah, Tamarah Koleala, Maria Kaius-Ome, Stephan Karl, Oriol Mitjà, Moses Laman, William Pomat, Quique Bassat

**Affiliations:** 1 ISGlobal, Hospital Clínic—Universitat de Barcelona, Barcelona, Spain; 2 Vector-Borne Diseases Unit, Papua New Guinea Institute of Medical Research, Madang, Papua New Guinea; 3 Lihir Malaria Elimination Programme, Lihir Island, Papua New Guinea; 4 Australian Institute of Tropical Health and Medicine, James Cook University, Cairns, Australia; 5 Fight Infectious Diseases Foundation, Hospital Germans Trias I Pujol, Badalona, Spain; 6 School of Medicine and Health Sciences, University of Papua New Guinea, Port Moresby, Papua New Guinea; 7 Centre for Health and Social Care Research (CESS), Faculty of Medicine, University of Vic - Central University of Catalonia (UVic - UCC), Vic, Catalonia Spain; 8 Lihir Medical Centre, International SOS, Lihir Island, Papua New Guinea; 9 ICREA, Pg. Lluís Companys 23, 08010 Barcelona, Spain; 10 Pediatrics Department, Hospital Sant Joan de Déu, Universitat de Barcelona, Esplugues, Barcelona, Spain; 11 Centro de Investigação Em Saúde de Manhiça (CISM), Maputo, Mozambique; 12 CIBER de Epidemiología y Salud Pública, Instituto de Salud Carlos III, Madrid, Spain

**Keywords:** Imported, Islands, Malaria, Plasmodium, Prevalence, Travellers

## Abstract

**Background:**

The Lihir Islands of Papua New Guinea host a mining operation that has resulted in a mine-impacted zone (MIZ) with reduced malaria transmission and a substantial influx of mine employees, informal cross-country traders, returning locals, and visitors. Prevalence of malaria parasites was assessed in travellers arriving on the Lihir Group of Islands to evaluate the risk of parasite importation.

**Methods:**

In 2018, a cross-sectional study at the airport and main wharf was conducted, targeting asymptomatic travellers who had been away from Lihir for at least 12 days. Microscopy, rapid diagnostic tests (RDTs), and quantitative PCR (qPCR) were used to determine *Plasmodium* parasite prevalence, employing logistic regression models to identify factors associated with qPCR positivity.

**Results:**

398 travellers arriving by plane and 402 arriving by boat were included. Both cohorts were significantly different. Mean age among travellers arriving by plane was 40.1 years (SD ± 10.1), 93% were male and 96% were employed at the mine. In contrast, among travellers arriving by boat, the mean age was 31.7 years (SD ± 14.0), 68% were male and 36% were employed at the mine. The prevalence of malaria infection among travellers arriving by plane was 1% by RDT and microscopy, and increased to 5% by qPCR. In contrast, those arriving by boat showed a prevalence of 8% by RDT and microscopy, and 17% by qPCR. Risk factors for infection were arriving by boat (OR 4.2; 95%CI 2.45,7.21), arriving from nearby provinces with high malaria incidence (OR 5.02; 95%CI 1.80, 14.01), and having been away from Lihir for 91 days or more (OR 4.15; 95%CI 2.58, 6.66). Being mine worker staying at the mine accommodation was related with less infection risk (OR 0.24; 95% CI 0.14, 0.43); while Lihirian residents returning from a trip, VFRs, or people with trading unrelated to mining had higher risks (p = 0.0066).

**Conclusions:**

Travellers arriving by boat faced increased risk of malaria infection than those arriving by plane. This subpopulation poses an import risk to the MIZ and the rest of Lihir Islands. Screening of high-risk groups at wharfs, and collaboration with nearby Islands, could sustain reduced transmission and facilitate malaria elimination strategies.

**Supplementary Information:**

The online version contains supplementary material available at 10.1186/s12936-023-04804-y.

## Background

In 2020, Papua New Guinea (PNG) reported over 750,000 confirmed malaria cases [1], accounting for 87% of all cases and 94% of all malaria-related deaths in the WHO’s Western Pacific region [2]. Malaria transmission in PNG exhibits geographical heterogeneity, with low endemicity in the high-altitude inland areas, and high transmission levels in the coastal areas [3]. The provinces most affected, including New Ireland, East and West New Britain, Sandaun (West Sepik), and Milne Bay, exhibit an incidence of over 200 yearly cases per 1,000 inhabitants [4]. The most prevalent *Plasmodium* species are *Plasmodium falciparum* and *Plasmodium vivax* with an overall prevalence by microscopy of 2.1% and 0.5%, respectively [5]; however, *Plasmodium malariae* and *Plasmodium ovale* are also present [6].

Lihir Islands, located in New Ireland province, host a gold mining operation of Newcrest Mining Ltd on their largest island, Aniolam. Due to the high malaria transmission rates in the area and the additional risks posed by deforestation and open-pit mining [7], the company collaborates in a public–private partnership to provide essential services such as electricity, improved sanitation, and healthcare services to its employees and part of the general public. This partnership with the local government aims to reduce the burden of communicable diseases in their operational setting. The Mine-Impacted Zone (MIZ) includes the communities surrounding the open pit, the main town Londolovit, the mining accommodation (a well-equipped housing camp within a 2 km^2^ area), and the airport, all located in the north-east of Aniolam. Similar to other public–private partnerships in regions with high endemicity of vector-borne diseases, efforts are also directed towards implementing vector control strategies [8]. Since 2006, Newcrest finances a vector control programme involving larval source management, such as drainage of potential smaller larval habitats and the application of larvicides to larger water bodies within the MIZ [9]. The rest of the Lihir Islands depend on universal coverage with long-lasting insecticidal nets (LLINs) for vector control, which are distributed free of charge through mass campaigns every three years. Despite achieving coverage rates of 97–98%, most of the population does not consistently use or maintain the LLINs over time [10].

In 2010, a cross-sectional study was conducted in Aniolam to evaluate malaria prevalence and the impact of the implemented vector control programmes [9]. While there was a marked reduction in malaria positive children detected by microscopy within the MIZ (from 31.5% in 2006 to 5.8% in 2010), the reduction was substantially smaller in the non-MIZ (from 34.9% in 2006 to 26.9% in 2010). Despite these efforts, malaria remains a common diagnosis, even among mine employees and contractors. In 2019, there were 784 malaria cases diagnosed among the workforce, of which 488 were identified in employees staying at the mine accommodation (unpublished, data provided by the Lihir Medical Center, funded by Newcrest and located within the MIZ). Of note, there are approximately 3000 mobile mine employees and contractors arriving by plane or boat from other areas within the same province or from other provinces. Beyond the mobile population related to mine activities, additional population movements occur between Aniolam and the neighbouring islands for trading purposes, along with an unspecified number of visiting friends and relatives (VFRs) between nearby islands and provinces.

Travellers are common sources of imported malaria. For instance, a prevalence survey in the low-burden island of Bioko, Equatorial Guinea, showed that malaria infection was highly related with having a history of travel to mainland Equatorial Guinea [11]. In PNG, the haplotypes of *P. vivax* isolates from different regions revealed patterns of transmission following major human migration routes, especially within the ‘Islands region’ of PNG [12]. These findings suggests that improved diagnosis and treatment of travellers could be critical for the success of malaria control and elimination strategies [13]. Even after achieving malaria elimination, the frequency of infected individuals entering non-endemic areas remains a primary risk factor for malaria re-establishment [14]. Cabo Verde serves as an example, where malaria resurged after interruption of local transmission in two occasions within the last 50 years, due to the imported infections by those travellers arriving from mainland Africa [15].

Herewith, a cross-sectional study was conducted to assess the prevalence of *Plasmodium* parasites in travellers arriving to the Lihir Islands by plane or boat, and to evaluate the importation risk they pose to the ongoing transmission within the MIZ and in the rest of Lihir. Parasite prevalence was determined using microscopy, RDT and quantitative PCR (qPCR), and factors associated with qPCR positivity were identified. Estimating the burden of malaria among travellers to Lihir can provide valuable insights for refining current control strategies and guiding the sustainability of future interventions aimed at malaria elimination on these islands.

## Methods

### Ethical considerations

This study obtained ethical clearance from the national ethical committee in PNG, the Medical Research Advisory Committee (PNG-MRAC), with MRAC No.18.07. Written informed consent was collected from all individuals in the study. Children under the age of 18 were verbally consented and parents or legal guardians signed the consent form on their behalf. Those participants unable to read and/or write were verbally consented and an impartial witness countersigned the consent form.

### Study setting

Lihir Islands are located 900 km northeast of Port Moresby in the New Ireland Province of PNG. Lihir consists of a group of four islands: Aniolam (the largest, with an area of 200 km^2^), and the smaller outer islands of Malie, Masahet, and Mahur. They are characterised by a tropical rainforest climate with extremely high precipitation figures all year round, a limited public health infrastructure, and a near-inaccessible geography in some areas. Londolovit is the main town and is the centre for most of the local business and those related with the mining activities. A population census, conducted between 2018 and 2020, estimated 26,528 inhabitants living in the Lihir Islands. Mining employees migrating from other parts of PNG, contribute to more than one third of the population on Aniolam [16].

### Study design and study population

Between the 30th of May and the 7th of December of 2018, a cross-sectional survey was conducted at the main points of entry for travellers, the Lihir airport and the Londolovit wharf. A non-random voluntary response sampling was used for recruitment. Individuals arriving at the wharf and airport were invited to visit the recruitment stand of the study through informative posters and oral announcements, using either one-on-one conversations or a loudhailer. Individuals, both male or female, aged 6 months or older; who were not residents of Lihir or had been away from the islands for more than 12 days; and did not report febrile syndrome for the 48 h before arrival were eligible for inclusion in the study. Exclusion criteria were unwillingness to provide informed consent or withdrawal of consent, as well as arrivals from a non-endemic country (travellers directly arriving from Australia, or arriving from any non-endemic country and who had only transited through Port Moresby airport).

### Data and sample collection

After signing the informed consent, a questionnaire was obtained from all participants, which included demographic and clinical data, information on LLIN usage, and mobility/travel information. Data collection was conducted using a paper questionnaire in the field, and subsequently, two data clerks independently entered the data into a database upon their return from the field. For each participant, a health practitioner performed a finger prick to collect blood drops for a malaria RDT (Malaria Pf/PAN Ag Combo RDT, Carestart™, USA), a blood slide for light microscopy examination, and 2 dry blood spots in filter paper for qPCR. All tests and materials were labelled with the participant identification number before sample collection. RDT was performed according to the manufacturer instructions. For the slide, two drops of blood were used for a thick and thin blood smear, using a clean spreader slide for the latter. Slides were air-dried in horizontal position and stored in slide boxes to transport to the local laboratory for staining and reading. Two blood spots of approximately 2.5 cm of diameter were fulfilled on filter paper, air-dried, and placed into separate zip-lock bags with silica gel. Once in the local laboratory, they were stored at -20 ºC until shipped to the IMR Vector-borne Diseases Unit in Madang (PNG) for further processing. Results of the RDT were immediately interpreted by a clinician who provided antimalarial treatment according to PNG treatment guidelines in case of a positive result.

### Laboratory procedures

Upon return from the field, only thin smear was fixed with methanol and blood slides were stained with 10% Giemsa during 10 min at the local laboratory following the MM-SOP-07A from the World Health Organization (WHO). Blood slides were examined under × 1000 magnification by two independent level 1–2 microscopists who had completed WHO quality assurance courses. A sample was considered negative after examining one hundred fields of view. When a parasite was observed, counts of white cells and parasites were conducted until 300 white cells were counted. The parasite density in parasites per μL was then calculated, assuming a white cell count of 8000 white cells/μL. The results for this first reading were cross-checked with the Institute of Medical Research (IMR) Vector-borne Diseases Unit at Madang (PNG). Any discrepancies were addressed at IMR with the involvement of a third WHO-certified level 1 microscopist.

DNA extraction was conducted at the IMR Vector borne Diseases Unit in Madang (PNG) using FavorPrepTM 96-well Genomic DNA kit (FAVORGEN^®^) and performed according to the manufacturer’s instructions to obtain genomic DNA from blood. Following DNA extraction, a generic qPCR ‘QMAL’ assay that amplifies a conserved region of the 18S rRNA gene was run on all samples [17]; and for all positive samples, a species-specific qPCR for *P. falciparum*, *P. vivax*, *P. malariae* and *P. ovale* were performed as previously described [18]. Finally, in cases where qPCR QMAL positive samples yielded negative results in the species-specific qPCR, ultra-sensitive qPCRs targeting Pf-varATS for *P. falciparum* and Pv-mtCOX1 for *P. vivax* were conducted [19, 20].

### Sample size and statistical analysis

The minimum sample size was 385 individuals arriving by plane and 385 individuals arriving by boat, considering a 5% precision and a 95% bilateral normal confidence interval (CI), and assuming an unknown prevalence of *Plasmodium* spp. carriage in travellers (50%).

Data were analysed using the statistical software STATA [21] and described as frequencies and mean (standard deviation, SD) for categorical and continuous variables, respectively. For typically skewed quantitative variables, the median and interquartile range (IQR) were also considered. Chi-squared test (or Fisher's exact test) and t-test were performed to assess differences between groups for categorical and continuous variables, respectively. Spearman’s rank correlations were used to estimate the association of continuous variables. Logistic regression models were used to determine the factors that were associated with qPCR positivity. For this model, the variable of origin or place stayed while being away from Lihir was stratified according to level of incidence following the 2019 National Department of Health report [3] as; low-incidence provinces (0 to 75 cases per 1,000 population: National Capital District, Bougainville, Hela, Enga, Western Highlands, Southern Highlands, Jiwaka, Chimbu, and Eastern Highlands); medium incidence provinces (76 to 225 cases per 1000 population: East Sepik, Western, Morobe, Central, Madang, Gulf and Milne Bay); and high-incidence provinces (> 225 cases per 1000 population: West Sepik, Oro, West New Britain, East New Britain, Manus and New Ireland). All significance levels were set at 0.05.

## Results

### Demographic and other characteristics of travellers arriving on Lihir

**Fig. 1:**
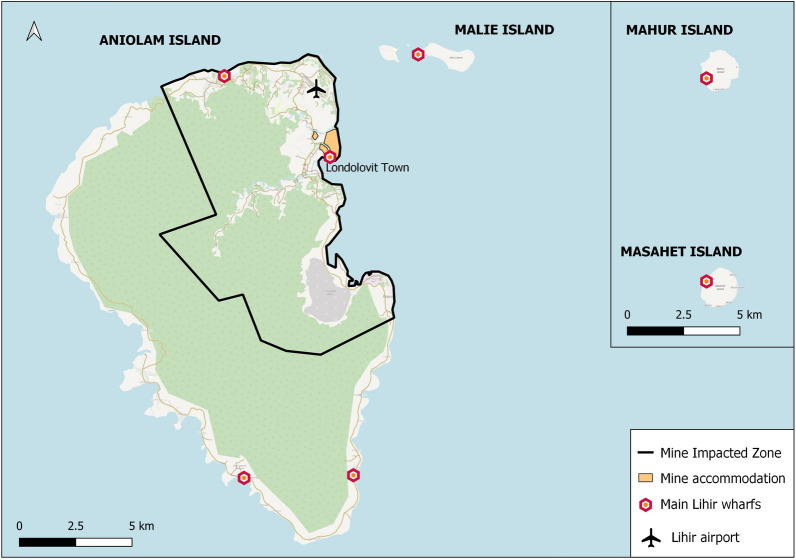
The Lihir Islands of Papua New Guinea. Legend: map showing the main points of entry for travellers, the limits of the mine impacted zone and the mine accommodation areas in the Lihir Islands

A total of 800 travellers arriving at the main points of entry in Lihir were recruited: the airport and the main wharf, located in Londolovit Town. Smaller wharfs outside the MIZ observe considerably fewer arrivals are were not included for sampling. The map in Fig. 1 shows the boundaries of the mine accommodation area, the MIZ, and the points of arrival for travellers in the Lihir Islands.

The study population comprised 398 participants arriving by plane and 402 participants arriving by boat. Table 1 presents the demographic and travel characteristics of both cohorts. These two groups were significantly different across nearly all recorded variables: sex, age, duration outside Lihir, purpose of the visit and place of stay while on Lihir (all p < 0.0001).

**Table 1:** Demographic, travel data and prevention measures used by the study participants

Variables		Arriving by plane (N = 398)	Arriving by boat (N = 402)	Total	p-value^*^
Demographic characteristics					
Sex	Male n (%)	369 (93)	273 (68)	642 (80)	< 0.0001
	Female n (%)	29 (7)	129 (32)	158 (20)	
Age (years) mean (SD)		40.1 (10.1)	31.7 (14.0)	35.9 (12.9)	< 0.0001
Age category	< 15 years old n (%)	0 (0)	47 (12)	47 (6)	< 0.0001
	15 to 29 years old n (%)	54 (14)	112 (28)	166 (21)	
	≥ 30 years old n (%)	344 (86)	243 (60)	587 (73)	
Travel information					
Time away from Lihir^a^ (days)	> 12 to 31 n (%)	348 (87)	213 (53)	561 (70)	< 0.0001
	32 to 90 n (%)	18 (5)	25 (6)	43 (5)	
	91 or more n (%)	32 (8)	163 (41)	195 (24)	
Place while on Lihir	Mine accommodation n (%)	395 (99)	140 (35)	535 (67)	< 0.0001
	Londolovit Town n (%)	3 (1)	94 (23)	97 (12)	
	Villages of Lihir n (%)	0 (0)	168 (42)	168 (21)	
Intention of the visit	Returning resident/VFR n (%)	0 (0)	122 (30)	122 (15)	< 0.0001
	Mine worker n (%)	383 (96)	144 (36)	527 (66)	
	Trading unrelated to mining n (%)	1 (0)	92 (23)	93 (12)	
	Other purpose n (%)	14 (4)	44 (11)	58 (7)	
Time intended to stay on Lihir (in days) ^b^ mean (SD)		20.2 (10.3)	22.1 (40.9)	21.1 (29.1)	0.3707
Malaria episodes and prevention measures					
Received malaria treatment last year	Yes n (%)	320 (80)	382 (95)	702 (88)	< 0.0001
Last malaria episode (in months) ^a^ mean (SD)		3.7 (0.7)	3.6 (0.9)	3.6 (0.8)	0.0888^c^
Frequency of sleeping under net while away from Lihir^a^	Never n (%)	217 (55)	221 (56)	438 (55)	0.0807
	Sometimes n (%)	93 (24)	108 (27)	201 (25)	
	Most nights n (%)	28 (7)	33 (8)	61 (8)	
	Always n (%)	57 (14)	34 (9)	91 (12)	
Slept under net while away from Lihir^a^	Yes n (%)	178 (45)	175 (44)	353 (45)	0.8834

LLIN = Long-lasting insecticidal net, SD = Standard Deviation, VFR = Visiting Friends and Relatives. ^a^ missingness < 1.1%, ^b^ n = 740 (7.5% missing); ^c^ Chi squared test; *significance level set at 0.05

Travellers arriving by plane were predominantly male (93%) with a mean age of 40.1 (SD ± 10.1) years, who were employed at the mine (96%) and intended to stay at the mine accommodation facilities (99%). Given the mine's standard 14-day work roster (14 days on Lihir, 12 days off), the majority of these fly-in fly-out employees (87%) had spent less than a month away from Lihir.

In contrast, travellers arriving by boat displayed greater diversity. While males still formed the majority, females (32%) and children (12%) were present, with a mean age of 31.7 (SD ± 14.0) years old. Their travel purposes also varied widely, with only 36% being mine workers. Many travellers arriving by boat were either Lihir residents returning home or engaging in VFRs (30%), trading or business outside the mine (23%) or visiting Lihir for other reasons (11%). Consequently, only 35% of this cohort planned to stay at the mine’s accommodation facilities, with most opting for either one of the Lihirian villages (42%) or Londolovit Town (23%). They also had spent longer periods outside Lihir prior to travelling, with 41% of them being away for 3 months or more.

There was a clear association between the place of stay on Lihir and the purpose of the visit, both in travellers arriving by plane (p = 0.0019) and in those arriving by boat (p < 0.0001). Conversely, despite variations in the place of stay and the intention of the visit, both cohorts shared a similar mean length of stay on Lihir (around 3-weeks).

### Origin of travellers, last malaria episode and use of LLINs

**Fig. 2:**
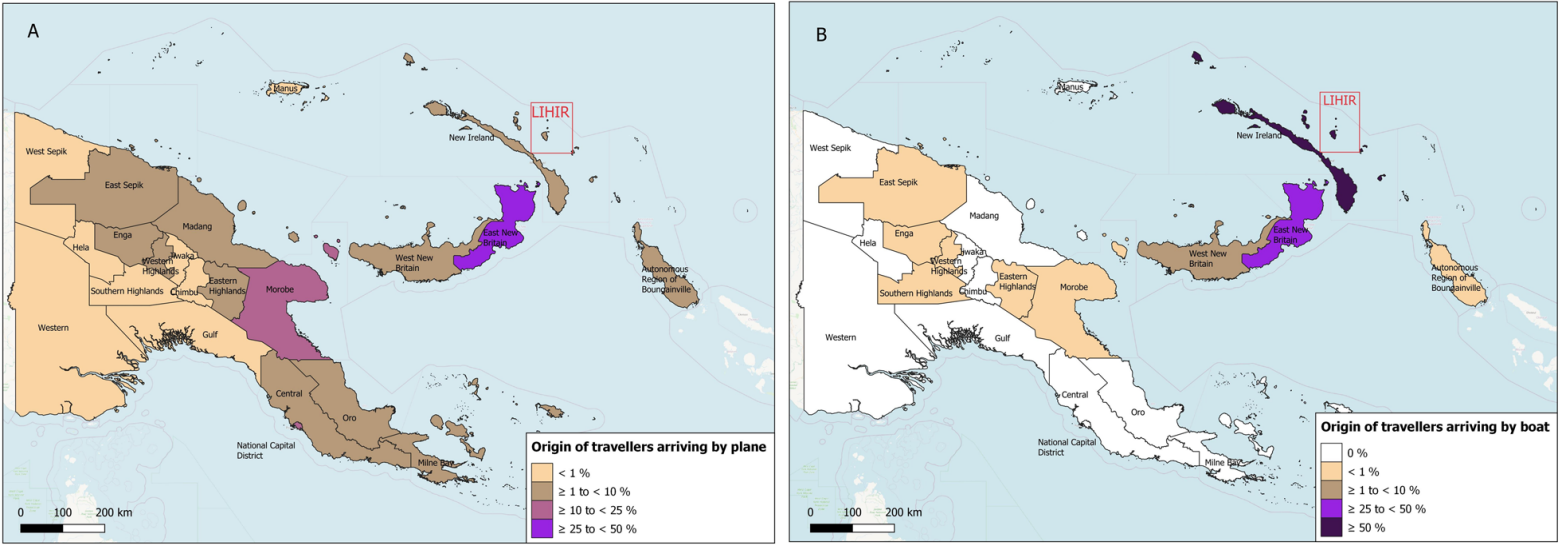
Origin of travellers arriving on the Lihir Islands. Legend: maps showing the percentage of travellers arriving to the Lihir Islands from each of the 22 provincial divisions of Papua New Guinea, in (**A**) the cohort of travellers arriving by plane; and in (**B**) the cohort of travellers arriving by boat

The origin of travellers arriving to Lihir differed significantly between those arriving by plane and those arriving by boat (p < 0.0001). Figure 2 illustrates the 22 provincial-level divisions of PNG and the origin of travellers arriving on Lihir by plane or boat. Travellers arriving by plane came from various provinces in PNG, including regions of high, moderate and low malaria transmission. Almost all PNG provinces were represented in this cohort, with East New Britain and the National Capital District being the most prominent, accounting for 26% and 19% of the travellers, respectively. In contrast, the majority of travellers arriving by boat came from the nearby islands of New Ireland (56%) or East New Britain (39%); where malaria transmission is high.

Despite the different origin of travellers in both cohorts, most reported having received a full course of antimalarial treatment during the preceding 12 months. However, this proportion was higher among travellers arriving by boat (95%) compared to those arriving by plane (80%) (p < 0.0001). The reported time of the last malaria episode was between 3 and 4 months prior to enrolment in the study for both cohorts. In addition, the use of LLIN was similar, with most travellers not utilizing them while being away from Lihir. No participant had fever, or any active symptom compatible with malaria upon recruitment.

### Prevalence of *Plasmodium* parasites in travellers arriving on Lihir

Regarding infection status of travellers arriving by plane, only 3 cases (1%) were positive by RDT and microscopy. Microscopy showed one case of *P. falciparum* infection and two of *P. vivax*. DNA extraction was possible in 396 of the 398 blood spots collected in the travellers arriving by plane. When using qPCR to detect *Plasmodium* parasites, 18 (5%) of these travellers were positive, 16 (4%) were male and 2 (7%) females. Most of the positive cases arrived from provinces where malaria transmission is high or moderate, including East New Britain (6), East Sepik (3), Morobe (3), New Ireland (2), and Western Province (1). Unfortunately, the amount and/or quality of the extracted DNA from these samples was not sufficient to successfully determine *Plasmodium* species in the subsequent specification reaction, with 77% of the positive qPCR samples yielding a negative result.

Conversely, among travellers arriving by boat, 33 (8%) were positive by RDT and microscopy. Microscopy showed 22 (67%) cases of *P. falciparum* infection, 5 (15%) of *P. vivax*, 1 (3%) of *P. malariae,* and 5 (15%) of mixed infections, which included *P. falciparum* and *P. vivax*. In contrast, RDT diagnosed 14 (42%) cases of *P. falciparum* infection, 10 (30%) of non-*P. falciparum* species, and 9 (27%) potential mixed infections. When using qPCR, 67 (17%) of travellers arriving by boat were positive; 43 (64%) were male and 24 (36%) were female. Most of them (98%) arrived from either New Ireland (43 individuals) or East New Britain (23 individuals), and 1 arrived from the Autonomous Region of Bougainville. In this cohort, DNA extraction was optimized and 63% of the positive qPCR samples yielded a positive result for the subsequent specification reaction. Among those, 21 were positive for *P. falciparum*, 6 for *P. vivax*, 2 for *P. malariae*, 11 for both *P. falciparum* and *P. vivax*, and 3 for *P. falciparum* and *P. malariae*. There were no *P. ovale* positive samples.

**Fig. 3:**
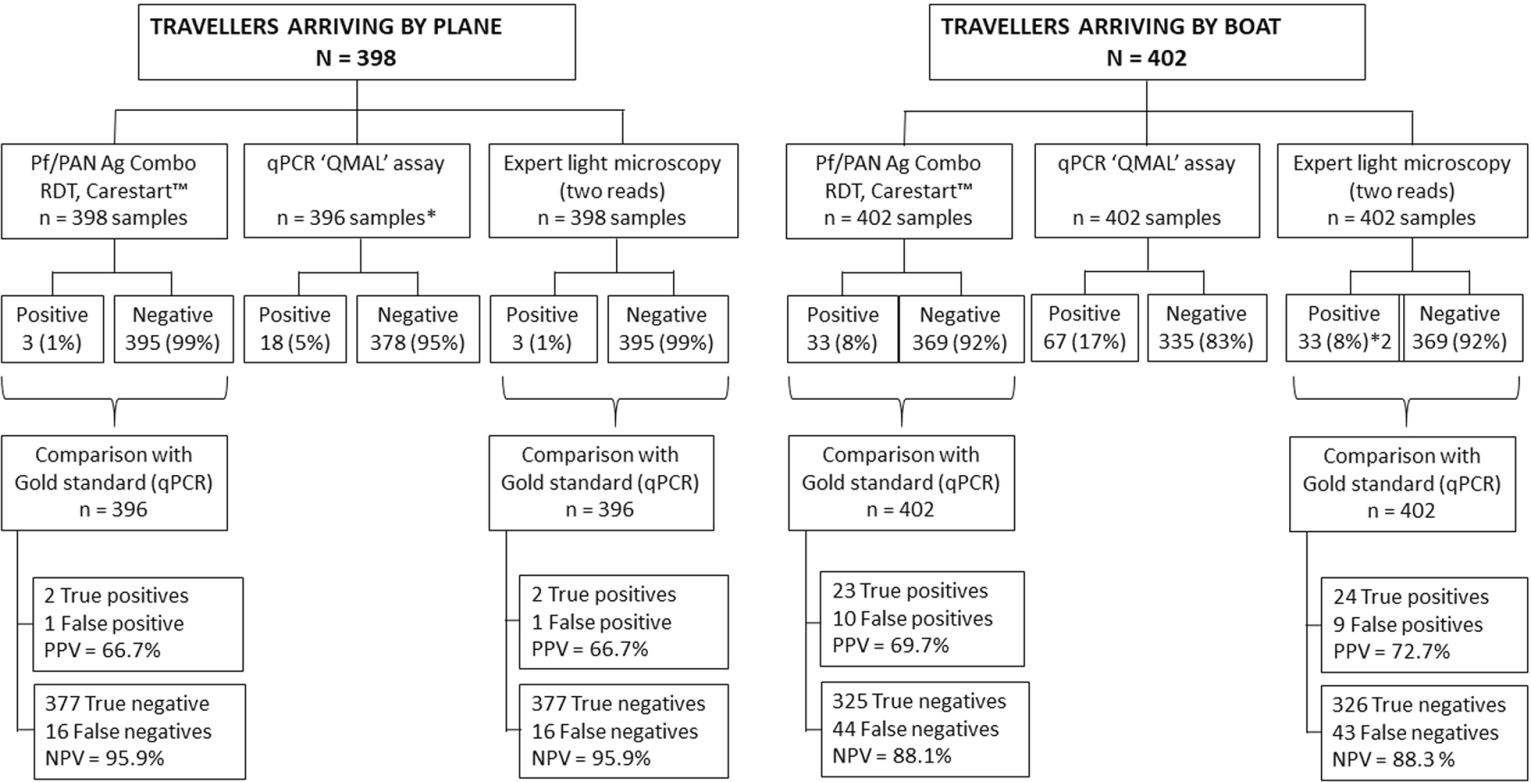
Results and accuracy of the malaria diagnostic tests in both cohorts of travellers. Legend: tests’ accuracy was calculated using qPCR as the reference test. PPV (positive predictive value) and NPV (negative predictive value) were calculated using the described prevalence in each cohort as the pre-test probability. *: DNA extraction was possible in 396 of the 398 blood spots collected; *2: although same number of positive samples by microscopy, they were not the same samples

A flowchart of the samples tested in each cohort by each of the techniques, including their results and the tests’ accuracy using qPCR as the reference test, is available in Fig. 3.

### Factors associated with malaria infection

The uni- and multivariate logistic regression models including both cohorts of travellers are shown in Table 2. In the univariate analysis, there was an increased risk of qPCR confirmed malaria infection among those arriving by boat (OR 4.2; 95% CI 2.45, 7.21), in those arriving from PNG provinces with high malaria incidence (OR 5.02; 95% CI 1.80, 14.01), and in individuals that had been away from Lihir more than 91 days (OR 4.15; 95% CI 2.58, 6.66). Being mine worker offered protection against carrying malaria parasites (OR 0.24; 95% CI 0.14, 0.43) compared to other intention of the visit such as Lihirian resident returning from a trip or VFRs, or people with intention to trading out of the mine business (OR 1.27; 95% CI 0.67, 2.43). Travellers intended to stay at Londolovit Town (OR 5.93; 95% CI 3.33, 10.56) or at the villages of Lihir (OR 3.24; 95% CI 1.88, 5.58) had higher risk compared to those planning to stay at the mine accommodation. None of the assessed travel characteristics showed a relationship with malaria infection detected by qPCR, except for the fact of being away from Lihir for more than 91 days (aOR 2.53; 95% CI 1.25, 5.10). Additionally, the univariate logistic regression model showed that being male (OR 0.52; 95% CI 0.31, 0.85) and age (OR 0.57; 95% CI 0.25, 1.30 for 15–29 years-old; OR 0.37; 95% CI 0.17, 0.78) being protective factors for infection; however, these findings could not be confirmed in the multivariate model. Finally, there was no association between having slept under LLIN while away from Lihir (OR 1.28; 95% CI 0.81, 2.02) and malaria infection.

**Table 2:** Logistic regression analyses for *Plasmodium* qPCR positive results in all the travellers

Variable	Univariate model		Multivariate model	
	OR (95% CI)	p-value	aOR (95% CI)^#^	p-value*
Sex				
Female	Reference group	0.0092	Reference group	0.6828
Male	0.52 (0.31, 0.85)		1.13 (0.62, 2.06)	
Age (years)				
< 15	Reference group	0.0191	Reference group	0.9384
15 to 29	0.57 (0.25, 1.30)		0.92 (0.38, 2.24)	
≥ 30	0.37 (0.17, 0.78)		0.87 (0.37, 2.01)	
Arriving by				
Plane	Reference group	< 0.0001	Reference group	0.5655
Boat	4.20 (2.45, 7.21)		1.69 (0.68, 4.21)	
Origin (arriving from)^a^				
Low incidence PNG provinces	Reference group	0.0004	Reference group	0.3703
Medium incidence PNG provinces	1.58 (0.43, 5.73)		1.74 (0.47, 6.50)	
High incidence PNG provinces	5.02 (1.80, 14.01)		2.34 (0.72, 7.62)	
Time away from Lihir (days)^b^				
> 12 to 31	Reference group	< 0.0001	Reference group	0.0308
32 to 90	1.45 (0.49, 4.28)		1.28 (0.40, 4.09)	
91 or more	4.15 (2.58, 6.66)		2.53 (1.25, 5.10)	
Place while on Lihir				
Mine accommodation	Reference group	< 0.0001	Reference group	0.1834
Londolovit Town	5.93 (3.33, 10.56)		1.80 (0.43, 7.48)	
Villages of Lihir	3.24 (1.88, 5.58)		1.00 (0.24, 4.17)	
Intention of the visit				
Returning resident/VFR	Reference group	< 0.0001	Reference group	0.4856
Mine worker	0.24 (0.14, 0.43)		0.82 (0.19, 3.45)	
Trading unrelated to mining	1.27 (0.67, 2.43)		1.04 (0.53, 2.04)	
Other purpose	0.45 (0.17, 1.16)		0.48 (0.17, 1.30)	
Slept under net while away from Lihir ^b^				
No	Reference group	0.2830	Reference group	0.6750
Yes	1.28 (0.81, 2.02)		1.11 (0.68, 1.82)	

LLIN = Long-lasting insecticidal net, aOR = adjusted odds ratio, OR = odds ratio, PNG = Papua New Guinea, VFR = Visiting Friends and Relatives. ^a^n = 777 (2.6% missing), ^b^missingness < 1.1%. *significance level set at 0.05. ^#^multivariate analysis conducted with 767 observations

To further explore factors associated with malaria infection and their respective risk, separate analyses for each cohort were conducted. For travellers arriving by plane, no variable was found to be significantly associated with testing positive by qPCR or any of the utilized techniques (Additional table, Additional file 1). In contrast, among travellers arriving by boat, a significant association was observed between testing positive by qPCR and the duration of time spent outside Lihir (p = 0.0003), the purpose of the visit (p = 0.0066), and the place of stay on Lihir (p = 0.0011) (Table 3). The univariate logistic regression model showed that travellers spent three months or more away from the Island had a threefold higher risk of malaria infection (OR 3.01; 95% CI 1.71, 5.30). Those travellers intending to stay at Londolovit town (OR 3.74; 95% CI 1.80, 7.74) or at the villages (OR 1.95; 95% CI 0.97, 3.94) had also higher risk of infection. On contrary, mine workers had less risk of infection (OR 0.39; 95% CI 0.19, 0.79). The only independent risk factor shown after the multivariate logistic analysis was the time spent outside Lihir, with aOR 2.35 (95%CI 1.07, 5.18) for those travellers being 91 days or more outside the Islands. There were no significant associations between sex, age, point of origin or having slept under LLIN, and malaria infection as detected by qPCR.

**Table 3:** Variables and their associations with Plasmodium qPCR positive results and logistic regression models for the travellers arriving by boat

Variable	qPCR positive n (%)	Association p-value^*^	Univariate OR (95% CI)	Multivariate aOR (95% CI)^#^
Sex				
Female	24 (19)	0.4736	Reference group	Reference group
Male	43 (16)		0.82 (0.47, 1.42)	1.34 (0.71, 2.54)
Age (years)				
< 15	10 (21)	0.4341	Reference group	Reference group
15 to 29	21 (19)		0.85 (0.37, 1.99)	1.01 (0.40, 2.56)
≥ 30	36 (15)		0.64 (0.29, 1.41)	0.88 (0.36, 2.13)
Origin (arriving from)^a^				
Low incidence provinces	0 (0)	0.3983	Reference group	Reference group
Medium incidence provinces	0 (0)		1 (-)	1 (-)
High incidence provinces	67 (17)		1.21 (0.14, 10.23)	1.99 (0.19, 17.27)
Time away from Lihir (days)^a^				
> 12 to 31	22 (10)	0.0003	Reference group	Reference group
32 to 90	3 (12)		1.18 (0.33, 4.28)	1.20 (0.29, 4.89)
91 or more	42 (26)		3.01 (1.71, 5.30)	2.35 (1.07, 5.18)
Place while on Lihir				
Mine accommodation	13 (9)	0.0011	Reference group	Reference group
Londolovit Town	26 (28)		3.74 (1.80, 7.74)	1.64 (0.28, 9.63)
Villages of Lihir	28 (17)		1.95 (0.97, 3.94)	0.87 (0.15, 5.07)
Intention of the visit				
Returning resident/VFR	25 (20)	0.0066	Reference group	Reference group
Mine worker	13 (9)		0.39 (0.19, 0.79)	0.59 (0.10, 3.50)
Trading unrelated to mining	23 (25)		1.29 (0.68, 2.46)	1.07 (0.54, 2.13)
Other purpose	6 (14)		0.61 (0.23, 1.61)	0.55 (0.20, 1.53)
Frequency of sleeping under net while away from Lihir^a^				
Never	32 (14)	0.6155	Reference group	Reference group
Some nights	20 (19)		1.34 (0.73, 2.48)	1.23 (0.65, 2.34)
Most of the nights	4 (12)		1.05 (0.38, 2.93)	0.72 (0.24, 2.19)
Always	7 (21)		2.13 (0.91, 4.97)	2.14 (0.84, 5.48)
Slept under net while away from Lihir ^a^				
No	32 (14)	0.1894	Reference group	Not considered for this analysis
Yes	34 (9)		1.42 (0.84, 2.42)	

aOR = adjusted odds ratio, OR = odds ratio, qPCR = quantitative polymerase chain reaction, VFR = visiting friends and relatives. ^a^ all missingness < 1.5%. *significance level set at 0.05. ^#^multivariate analysis conducted with 393 observations

## Discussion

This study characterized the prevalence of malaria parasites in travellers arriving on Lihir Islands to estimate the risk of malaria importation. This study revealed a substantial four-fold increase in the risk of malaria infection among travellers arriving by boat compared to those arriving by plane. Demographic and travel characteristics of the two cohorts had distinct profiles that likely contribute to differing risks of malaria infection.

The travellers arriving by plane predominantly consisted of adult male mine workers, who were on a 14-day work roster and stayed at the mine accommodation facilities. This group exhibited a lower prevalence of malaria parasites, likely due to their diverse origins from provinces with varying transmission intensities, and possibly in relation to better protection practices related to improved living conditions. In contrast, the cohort of travellers arriving by boat exhibited greater diversity, with the presence of females and children, and were mostly locals or residents in neighbouring islands. This group had a notably higher prevalence of malaria parasites, indicating increased risk of infection associated with their demographic and travel characteristics, including longer stays away from Lihir. Notably, qPCR-detected infections in travellers arriving by boat (17%) closely resembled the mean prevalence in the local Lihirian population (15%) found in a parallel study in 2019 (Millat-Martinez et al., under revision). This high number of asymptomatic individuals (both local and mobile) carrying malaria parasites likely sustain the ongoing transmission in Lihir Islands [22]. In contrast, malaria prevalence in travellers arriving by plane (5%) was lower than any prevalence found in Lihir Islands.

Furthermore, when looking for risk factors associated with malaria infection in the whole cohort and the cohort of travellers arriving by boat, the only independent risk factor identified was an extended absence from Lihir, specifically over 91 days. This further supports their increased exposure and subsequent elevated infection rates. Although arriving from high incidence PNG provinces emerged as a risk for infection, there was no independent association between point of origin and malaria infection, probably because most of the travellers arriving by boat arrived from high incidence provinces. Interestingly, the univariate logistic regression analysis in the whole cohort and in the cohort of travellers arriving by boat showed an association between infection and visit intent. Lihirian residents returning home, VFRs and traders had higher risk of infection compared to mine employees and contractors. These findings contrast with other mining settings, especially illegal operations, where mine workers presented higher malaria burden than the indigenous population [23, 24]. This divergence might be attributed to the relatively higher socioeconomic status, better living conditions, sanitation, and awareness of malaria prevention among Lihir mine employees and contractors. Stay location in Lihir also influenced infection risk, as mine workers exclusively used the well-conditioned mine accommodations within the MIZ, where moreover *Anopheles* biting intensity and inoculation rates are highly reduced compared to the rest of Lihir (Millat-Martinez et al., under revision).

Despite differing malaria parasite prevalence and province origins, the large majority of both cohorts had experienced at least one malaria episode in the last 12 months. A potential explanation is that travellers from low transmission provinces could have been exposed during their work roster in Lihir; while those coming from nearby islands might have been infected locally or in Lihir. In addition, PNG’s population, having been exposed to *P. vivax,* is likely to be carriers of hypnozoites, contributing to a high annual relapse burden [25]. Interestingly, both cohorts of travellers showed a concerning low use of LLIN while outside Lihir. However, it exceeded the local use described in 2018, where only 13.6% of people in Lihir reported having slept under a LLIN years after the mass distribution campaign in 2016 [10]. On the other hand, LLIN usage away from Lihir did not correlate with malaria infection. This may be explained by the exhibited decline in bioefficacy of distributed nets in PNG [26, 27], or by the early biting of infected *Anopheles* when people are still engaged in outdoor activities [28]. Hence, ensuring LLIN quality and enhancing its use should be a priority, as well as developing innovative vector control tools adapted to this particular mosquito behaviour.

This study included participants who had been away from Lihir for a minimum of 12 days, with the aim of capturing mine workers following the 14-day on-site and 12-day off-site work roster. Notably, while *P. falciparum* typically manifest an incubation period of 9 to 14 days*,* other species can have longer incubation periods [29]. Additionally, in PNG up to 80% of *P. vivax* active cases are estimated to be relapses [30], usually relapsing 3–6 weeks apart from the primary infection [31]. Thus, despite not being able to ensure that all infections were contracted away from Lihir, these infections represent the parasite load that could potentially contribute to local transmission after travellers’ arrival. Hence, a limitation of this study is that pattern of travel while outside Lihir was not comprehensively described, nor parasite genotyping used to confirm whether and infection was locally acquired or imported (and from where). On the other hand, another limitation of this study is that 77% of the qPCR positive samples from the cohort of travellers arriving by plane and 37% from those arriving by boat yielded negative results for the species specification reaction. The extracted DNA was of low amount and quality, which may be explained by possible disruptions in the cold chain interfering with DNA stability [32], or by degradation, considering that DNA extraction occurred 3 years after sample collection.

In the last decade, Newcrest Mining ltd has considered the possibility of malaria elimination in the Lihir Islands by implementing a pilot programme that integrates diverse strategies to reduce the malaria burden and ultimately halt local transmission. However, the amount of imported malaria parasites could challenge these efforts [33]. For instance, in Zanzibar, Tanzania, a stochastic model studying local transmission estimated up to 18% of imported cases and 25% of introduced (locally transmitted after an imported case), which noteworthy contributed to the local malaria burden and transmission [34]. Targeting travellers in low and moderate transmission settings have the potential to reduce local malaria burden, if encompassed with robust surveillance and response systems, intensive vector control, awareness programs, healthcare training, and frequent epidemiological and entomological monitoring [35, 36]. In Lihir, the primary risk of importing malaria stems from individuals arriving by boat, particularly returning residents, VFRs, and traders. Strategies to reduce infections in these groups while travelling to other PNG provinces of high and moderate malaria transmission could be considered, including education campaigns to improve the use of LLINs while abroad[13].

In islands regions like Lihir, where entry points are well identified, an effective approach for avoiding transmission of imported cases involves integrating passive and active case detection with travellers’ test and treat at borders [13, 37]. In accordance to other prevalence studies in PNG [38, 39], this study found a substantial number of submicroscopic infections. Ultra-sensitive RDTs and loop-mediated isothermal amplification could offer potential solutions for tackling these infections in travellers [40, 41], albeit with substantial limiting factors to consider during implementation. Hence, other approaches may be more feasible in Lihir. For instance, in Sri Lanka, two screening strategies for travellers based on standard RDTs have proven effective: testing upon arrival followed by a repeat test within two weeks [42], and targeting high-risk travellers [43].

Finally, islands do not have a guaranteed success in control and elimination programs [44], and collaboration with neighbouring areas is just as essential as it is in settings with land borders adjacent to endemic areas [45, 46]. Newcrest Mining Ltd's engagement with local and provincial governments, extending strategies beyond the MIZ, could yield sustainable, cost-effective results in curbing malaria amidst a mobile population in Lihir Islands.

## Conclusions

Travellers arriving by boat to Lihir Islands exhibit a significantly higher risk of malaria infection compared to those arriving by plane, thus posing a higher risk for parasite importation. Lihirian residents returning home, VFRs, and traders face a heightened risk for malaria infection in contrast to mine employees. Implementing screenings among high-risk travellers arriving by boat could potentially prevent transmission from importation. In the long-term, particularly if malaria transmission decreases across the Lihir Islands, improved control endeavours could greatly benefit from integrating actions in the neighbouring islands. Failing to proactively address imported malaria cases among travellers to the island could pose challenges to ensuring the sustainability and impact of malaria control or elimination efforts.

### Supplementary Information

**Additional file 1: Table S1.** Variables and their associations with Plasmodium qPCR positive results and logistic regression models for the cohort of travellers arriving by plane.

## Data Availability

The datasets used and/or analysed during the current study are available from the corresponding author on reasonable request.

## Abbreviations

CI: Confidence interval
IMR: Institute for Medical Research
LLIN: Long-lasting insecticidal net
MIZ: Mine-impacted zone
MRAC: Medical Research Advisory Committee
PNG: Papua New Guinea
qPCR: Quantitative Polymerase Chain Reaction
RDT: Rapid diagnostic test
SD: Standard deviation
VFR: Visiting friends and relatives
WHO: World Health Organization
