# Supplementary material for: A cross-sectional study to ascertain malaria prevalence among asymptomatic travellers arriving on the Lihir Group of Islands, Papua New Guinea: implications for elimination efforts
Source: Malar J. 2023 Nov 29;22:364. doi: 10.1186/s12936-023-04804-y (PMC10688477; doi:10.1186/s12936-023-04804-y)
Supplement: Supplementary file 1 — Additional file 1: Table S1. Variables and their associations with Plasmodium qPCR positive results and logistic regression models for the cohort of travellers arriving by plane. [file 12936_2023_4804_MOESM1_ESM.docx]

**ADDITIONAL FILE 1**

**AdditionalTable 1. Variables and their associations with Plasmodium qPCR positive results and logistic regression models for the cohort of travellers arriving by plane.**

| **Variable** | | **qPCR positive**  **n (%)** | **Association p-value*** | **Univariate**  **OR (95 % CI)** | **Multivariate**  **aOR (95 % CI)^#^** |
| --- | --- | --- | --- | --- | --- |
| Sex | Female | 2 (7) | 0.6324 | reference group | reference group |
|  | Male | 16 (4) |  | 0.62 (0.13, 2.82) | 0.54 (0.11, 2.66) |
| Age (years) | 15 to 29 | 1 (2) | 0.4876 | reference group | reference group |
|  | ≥30 | 17 (5) |  | 2.77 (0.36, 21.27) | 2.20 (0.28, 17.37) |
| Origin (arriving from)^a^ | Low incidence PNG provinces | 3 (2) | 0.2964 | reference group | reference group |
|  | Medium incidence PNG provinces | 7 (6) |  | 2.02 (0.49, 8.26) | 2.15 (0.49, 9.42) |
|  | High incidence PNG provinces | 8 (6) |  | 2.48 (0.64, 9.56) | 2.62 (0.65, 10.61) |
| Time away from Lihir (days)^b^ | >12 to 31 | 15 (4) | 0.6004 | reference group | reference group |
|  | 32 to 90 | 1 (6) |  | 1.30 (0.16, 10.44) | 1.43 (0.17, 12.07) |
|  | 91 or more | 2 (6) |  | 1.53 (0.33, 7.00) | 3.19 (0.62, 16.35) |
| Place while on Lihir | Mine accommodation | 18 (5) | 1 | reference group | not considered for this analysis |
|  | Londolovit Town | 0 (0) |  | 1 (-) |  |
| Intention of the visit^c^ | Mine worker | 18 (5) | 1 | reference group | not considered for this analysis |
|  | Trading unrelated to mining | 0 (0) |  | 1 (-) |  |
|  | Other purpose | 0 (0) |  | 1 (-) |  |
| Frequency of sleeping under net while away from Lihir ^d^ | Never | 10 (5) | 0.8312 | reference group | reference group |
|  | Some nights | 3 (3) |  | 0.69 (0.19, 2.57) | 0.65 (0.17, 2.50) |
|  | Most of the nights | 2 (7) |  | 1.59 (0.33, 7.67) | 1.31 (0.25, 6.83) |
|  | Always | 3 (5) |  | 1.19 (0.32, 4.50) | 0.63 (0.12, 3.29) |
| Slept under net while away of Lihir ^d^ | No | 10 (5) | 0.9764 | reference group | not considered for this analysis |
|  | Yes | 8 (4) |  | 0.99 (0.38, 2.55) |  |

Abbreviations: aOR = adjusted odds ratio, OR = odds ratio, PNG = Papua New Guinea, qPCR = quantitative polymerase chain reaction. ^a^n = 376 (5.0% missing), ^b^n = 392 (1.0 % missing), ^c^n = 381 (3.8% missing), ^d^n = 393 (0.7% missing). *significance level set at 0.05. ^#^multivariate analysis conducted with 358 observations.
